# Urodynamics assessment in patients with multiple sclerosis

**DOI:** 10.1002/bco2.70208

**Published:** 2026-04-28

**Authors:** Nicholas Faure Walker, Alexandra Bacon, Manisha Teji, Eskinder Solomon, Arun Sahai, Ross Stephens, Emmanuel Okpii, Charlotte Dunford, Sarah Wood, Emily Speck, Ruth Doherty, Angie Rantell, George Araklitis, Lydia Lilis, Tharani Nitkunan, Michelle Carey, Suzanne Biers, Brendan Berry, Mahreen Pakzad, Hashim Hashim

**Affiliations:** ^1^ Epsom and St Helier University Hospitals NHS Trust Epsom UK; ^2^ King's College London London UK; ^3^ Bristol Urological Institute Bristol UK; ^4^ North Bristol NHS Trust Bristol UK; ^5^ Guy's and St Thomas NHS Foundation Trust London UK; ^6^ Norfolk and Norwich University Hospitals NHS Foundation Trust Norwich UK; ^7^ King's College Hospital NHS Foundation Trust London UK; ^8^ Brunel University Uxbridge UK; ^9^ Cambridge University Hospitals NHS Foundation Trust Cambridge UK; ^10^ University College London Hospitals NHS Foundation Trust London UK

**Keywords:** detrusor overactivity, multiple sclerosis, neurogenic bladder, overactive bladder, urodynamics

## Abstract

**Objectives:**

The aim of this study is to review initial urodynamics (UDS) findings in patients with multiple sclerosis (MS) related lower urinary tract symptoms, establish where UDS happen in treatment pathways and to evaluate the benefit of video over standard UDS.

**Patients and Methods:**

OnaBotulinumToxin A (BTX‐A) naïve, MS patients who had undergone UDS were identified from prospectively maintained databases from UK hospitals.

**Results:**

One hundred fifty‐seven patients were identified from seven UK departments. One (0.9%) female patient and 3 (6.7%) males had end fill pressures > 40 cmH_2_O. No female and two (5.1%) males showed vesico‐ureteric reflux. Of female patients reporting pure overactive bladder (OAB) symptoms, 61 (67.2%) showed neurogenic detrusor overactivity (NDO), 10 (11.1%) showed loss of compliance (LoC) and 28 (31.1%) showed no NDO or LoC. Following UDS, 134 (85.4%) patients underwent management changes: 61 (72.6%) females and 25 (78.1%) males were offered non‐medical treatment, oral or topical medication; 30 (30.9%) females and 7 (18.9%) males were advised to start self‐catheterisation; 16 (19.0%) females and 4 (12.5%) males were offered intra‐vesical BTX‐A; two (2.4%) females were offered SUI surgery and one (3.1%) male was offered bladder outflow obstruction surgery.

**Conclusion:**

Many patients underwent UDS prior to conservative and medical management, which is earlier than most guidelines recommend, potentially delaying non‐invasive treatment. This study supports the need for VUDS at initial assessment of men with MS. Standard UDS suffice in women with MS and pure OAB symptoms unresponsive to conservative measures and oral medication unless they have abnormal renal function, hydronephrosis or significant post void volumes.

## 1 INTRODUCTION

Multiple sclerosis (MS) affected 152 000 people in the United Kingdom in 2022 and is the commonest cause of neurological disability in young people.^1^, ^2^, ^3^ Its worldwide prevalence is increasing.^2^ Urinary symptoms are reported by up to 99% of patients with MS during the course of the disease.^4^ Lower urinary tract symptoms (LUTS) including urinary frequency are reported by 25%–99%, urgency by 32%–86%, urgency urinary incontinence (UUI) by 19%–80% and urinary retention (UR) in 8.3%–73.8% of patients.^4^ Studies that have investigated urodynamics (UDS) findings in patients with MS have identified neurogenic detrusor overactivity (NDO) in 22.5%–99%, detrusor underactivity (DU) in 0%–40%, a loss of compliance in 2%–10.3% and detrusor sphincter dyssynergia (DSD) in 5%–82%.^4^

The UK consensus on the management of the bladder in MS provides very helpful management algorithms to guide management of urinary symptoms.^5^ The initial management involves testing for urinary tract infection (UTI) and starting intermittent self‐catheterisation (ISC) if there is a post‐void residual volume of over 100 mL. Following this, anticholinergic medications are recommended. Anticholinergics are however associated with multiple side effects including dry mouth and constipation, and more recently concerns have been highlighted regarding increased risks of cardiovascular disease and dementia.^6^, ^7^ The latter side effect is of concern in a population already at risk of cognitive dysfunction due to MS itself.^8^ Since the publication of this consensus document, Mirabegron and Vibegron, beta 3 adrenoceptor agonists, have also been used as an alternative or in addition to anticholinergic medication.^9^, ^10^

If a patient has refractory overactive bladder syndrome (rOAB) despite oral medications, urodynamic investigations are recommended in patients who wish to undergo further interventions (Table 1). The UK consensus does acknowledge that upper tract dilatation is uncommon in patients with MS and that the ‘benefit of urodynamics does not invariably warrant the intrusive nature of the study and the risks associated’.^5^ This is in contrast to a French guideline proposal, published in 2007, which proposes UDS in any patient with MS who presents with urinary symptoms.^4^ The 2012 National Institute for Health and Care excellence (NICE) guidelines (updated in 2023) on urinary incontinence in neurological disease do not recommend UDS as a baseline investigation for patients with MS as they are at low risk for upper tract deterioration.^12^ The NICE guidelines do however recommend UDS prior to surgical intervention including intra‐detrusor BTX‐A injections for rOAB. This may partly be due to licensing of BTX‐A in neurogenic detrusor overactivity, which is a urodynamic diagnosis. Since the publication of these documents, El Helou et al. published the results of a randomised trial evaluating the benefit of baseline invasive UDS in addition to uroflowmetry in patients with lower urinary tract symptoms (LUTS) and MS.^14^ The addition of invasive UDS at initial evaluation did not lead to improved symptom or quality of life scores at 6 months when compared to patients who underwent uroflowmetry alone. Following assessment in El Helou et al.'s study, patients were most commonly started on anticholinergics. No patients received BTX‐A injections. The 2024 European Association of Urology (EAU) guidelines on neuro‐urology recommend VUDS at baseline and follow‐up in patients with neurogenic lower urinary tract dysfunction (NLUTD) but do not make specific recommendations for patients with MS.^11^ The AUA/SUFU guidelines on NLUTD from 2021 do not recommend UDS for the initial assessment of a patient with low risk NLUTD and do recommend multichannel UDS, renal function assessment and upper tract imaging in those with unknown risk LUTD.^13^

**TABLE 1:** Guidelines recommendations of urodynamic assessment in patients with MS.

Guidelines	Year	Recommendations for UDS at baseline
UK consensus on the management of the bladder in multiple sclerosis^5^	2009	For those with *refractory urinary urgency and incontinence* not responding to conservative measures and oral medications, and who are considering further surgical or intravesical treatment Video UDS for women with stress incontinence considering surgical treatment
International Francophone Neuro‐Urological expert study group recommendations for the neurogenic bladder in MS^4^	2007	Recommended as part of initial assessment of patient with lower urinary tract symptoms
EAU Guidelines on Neuro‐Urology^11^	2024	VUDS recommended at baseline for all patients with neurological disease
NICE guidelines of urinary incontinence in neurological disease^12^	2012	Not recommended in initial assessment of patients with MS Recommended prior to surgical or intravesical treatment in patients with MS
AUA/SUFU guidelines on neurogenic lower urinary tract dysfunction^13^	2021	UDS only recommended at baseline if the patient has unknown risk of upper tract deterioration. Not recommended in patients with low risk NLUTD

The UK consensus on the management of the bladder in MS and the 2012 NICE guidelines on urinary incontinence in neurological disease recommend intra‐detrusor BTX‐A injections in patients who do not respond sufficiently or do not tolerate antimuscarinic mediation.^5^, ^12^, ^15^ Sacral neuromodulation (SNM) is recommended in patients who do not respond sufficiently to BTX‐A injections by the 2009 UK consensus.^5^ The 2023 EAU guidelines mention SNM but do not make recommendations for its use in patients with neurological disease owing to the lack of randomised trials.^15^ SNM is not mentioned by the 2012 NICE guidelines,^12^ or in the 2023 update. A recent French study reports that in their cohort of patients with MS with nOAB, SNM exhibits clinical efficacy comparable to that observed in the non‐neurological population.^16^ The outcomes from NEMISIS are anticipated: This is a multicentre, randomised controlled trial to test the hypothesis that SNM has a therapeutic benefit in neurogenic overactive bladder caused by multiple sclerosis.^17^

This study aims to review the initial UDS findings in patients with MS related LUTS in the United Kingdom, establish where UDS are happening in the treatment pathway of MS related LUTS and to evaluate the benefit of video UDS over standard UDS.

## 2 PATIENTS (OR MATERIALS) AND METHODS

A cross‐sectional study of the initial UDS in patients with MS identified from prospectively maintained databases of seven UK hospitals was performed between January 2020 and November 2023. Patients all had confirmed diagnoses of MS and had been referred to urology departments for further management of LUTS. Patients without a diagnosis of MS, aged under 18 or who had had previous UDS were excluded. Baseline demographic data including sex and age, catheterisation status, the presence of OAB, UUI, stress urinary incontinence (SUI) and medication history were recorded. The International Continence Society (ICS) terminology^18^ on lower urinary tract dysfunction (LUTD) was used throughout and UDS were performed in accordance with ICS and UK continence society (UKCS) standards.^19^, ^20^

Data were collected and analysed using Microsoft Excel version 16.83 and IBM SPSS Statistics version 29.0.2.0.^20^ The study was registered locally as an audit (reference NFW003). Results were expressed in means with standard deviation (SD) for normally distributed data and medians with ranges of non‐normally distributed data. Student *t*‐tests were used to compare mean values and the Wilcoxon rank sum tests for median values.

## 3 RESULTS

One hundred fifty‐seven patients were identified from seven UK departments. VUDS were performed in 94 (84.7%) females and 40 (87.0%) males, and standard UDS were performed in 17 (15.3%) females and 6 (13.0%) males between January 2020 and November 2023. Baseline patient data at the time of UDS are shown in Table 2.

**TABLE 2:** Baseline data.

	*n*	Median age/y (range)	IDC	ISC	OAB	UUI	SUI	Tried AC	Tried B3A	No previous AC or B3A
F	111 (70.7%)	54.0 (23.6–84.0)	8 (7.2%)	21 (18.9%)	95 (86.4%)	86 (78.9%)	46 (51.1%)	33 (30.0%)	30 (27.3%)	64 (57.7%)
M	46 (29.3%)	58.0 (25.0–84.3)	2 (1.8%)	10 (21.7%)	36 (78.3%)	25 (56.8%)	2 (4.5%)	6 (13.6%)	3 (6.8%)	36 (78.3%)
*p*		0.531	0.507	0.596	0.211	0.005	0.000	0.035	0.191	0.053
T	157		10 (9.0%)	31 (19.7%)	131 (84.0%)	111 (72.5%)	48 (35.8%)	39 (25.3%)	33 (21.4%)	100 (63.7%)

Abbreviations: AC = anticholinergics; B3A = beta‐3 agonists; F = female; IDC = indwelling catheter; ISC = intermittent self‐catheterisation; M = male; OAB = over active bladder; SUI = stress urinary incontinence; T = total; UUI = urgency urinary incontinence.

Urodynamic findings are shown in Table 3. Of patients reporting OAB, 61 (67.2%) females showed NDO, 10 females (11.1%) showed LoC, 28 (31.1%) females showed no NDO or LoC, 29 (80.6%) males showed NDO, two (5.6%) males showed LoC and six (16.7%) showed no NDO or LoC. Of the 5 (4.5%) women who reported pure SUI, all five showed urodynamic SUI without any NDO.

**TABLE 3:** Urodynamic findings.

	*n*	NDO	Poor compliance (<20 mL/cmH_2_O)	EFP > 40 cmH_2_O	MCC/mL (SD)	Reflux	USUI	PVR/mL (range)	Qmax/mL/s
F	111 (70.7%)	70 (63.1%)	11 (9.9%)	1 (0.9%)	368 (185)	0 (0%)	28 (26.9%)	135.2 (0–800)	14.7
M	46 (29.3%)	35 (76.1%)	5 (10.9%)	3 (6.7%)	391 (186)	2 (5.1%)	0 (0%)	161.4 (0–489)	7.80
*p*		0.116	0.347	0.042	0.203	0.088	0.00	0.023	0.000
T	157	105 (66.9%)	19 (12.1%)	4 (3.3%)	375	2 (2.0%)	28 (19.0%)	101.5 (0–800)	12.4

Abbreviations: EFP = end fill pressure; F = female; M = male; MCC = maximum cystometric capacity; NDO = neurogenic detrusor overactivity; poor compliance = <20–40 mL/cmH_2_O; PVR = post‐void residual; Qmax = maximum flow rate; T = total; USUI = urodynamic stress urinary incontinence.

Of the 69 (62.1%) female patients who had completed the voiding phase of UDS, the mean Qmax was 14.6 mL/s (range 2–54 mL/s). The mean PDetQmax was 27.3 cmH_2_O (range 0–85 cmH_2_O); 21 (30.4%) females had a PDetQmax less than 15 mL/s; 48 (69.6%) had a PDetQmax of 15 mL/s or over and 27 (39.1%) had PDetQmax over 15 cmH_2_O together with a Qmax of less than 15 mL/s.

Outcomes following urodynamics are shown in Table 4. Overall, following UDS, 134 (85.4%) patients underwent change in management: 31 women were offered conservative treatment (13 no change in treatment, six pelvic floor muscle exercises, six bladder training and six fluid and lifestyle advice). Conservative treatment was offered to 13 men (10 no change in treatment and three fluid and lifestyle advice). Sixty‐one (55.0%) women and 25 (54.3%) men were offered oral medication, topical treatment or non‐medical treatment such as pelvic floor physiotherapy, fluid and lifestyle advice; 30 (27.0%) women and seven (15.2%) men were advised to start ISC; 16 (14.4%) women and four (8.7%) men were offered intra‐vesical BTX‐A; four (3.6%) women were offered sacral neuromodulation (SNM) or posterior tibial nerve stimulation (PTNS); two (1.8%) women were offered surgery for SUI and two (4.3%) men were offered surgery for bladder outflow obstruction (BOO).

**TABLE 4:** Outcomes after urodynamics.

	*n*	No change in treatment	PFME	Bladder training	Fluid/lifestyle advice	Conservative–total
F	111 (70.7%)	13 (11.7%)	6 (5.4%)	6 (5.4%)	6 (5.4%)	31 (27.9%)
M	46 (29.3%)	10 (21.7%)	0 (0%)	0 (0%)	3 (6.5%)	13 (28.3%)
T	157	23 (14.6%)S	6 (3.8%)	6 (3.8%)	9 (5.7%)	44 (28.0%)

	*n*	Topical oestrogen	Alpha blocker	AC or B3A	Total–medical
F	111 (70.7%)	1 (0.9%)	2 (1.8%)	27 (24.3%)	30 (27.0%)
M	46 (29.3%)	0 (0%)	4 (8.7%)	8 (17.4%)	12 (26.1%)
T	157	1 (0.6%)	6 (3.8%)	35 (22.3%)	42 (26.8%)

	*n*	Start ISC	BTX‐A	PTNS	SNM	SPC	BOO surgery	SUI surgery
F	111 (70.7%)	30 (27.0%)	16 (14.4%)	2 (1.8%)	2 (1.8%)	5 (4.5%)	‐	2 (1.8%)
M	46 (29.3%)	7 (15.2%)	4 (8.7%)	‐	‐	2 (4.3%)	2 (4.3%)	‐
T	157	37 (23.6%)	20 (12.7%)	2 (1.8%)	2 (1.8%)	7 (4.5%)	2 (4.3%)	2 (1.8%)

Abbreviations: AC = anticholinergics; B3A = beta 3 agonist; BOO = bladder outflow obstruction; BTX‐A = botulinum toxin‐A; F = female; ISC = intermittent self‐catheterisation; M = male; PFME = pelvic floor muscle exercises; PTNS = posterior tibial nerve stimulation; SNM = sacral neuromodulation; SPC = suprapubic catheter; SUI = stress urinary incontinence; T = total.

A total of 57.7% of women underwent UDS before they had tried an anticholinergic or beta 3 adrenoceptor agonist. Outcomes based on urodynamic findings are shown in Table 5: Of 70 women and 35 men with NDO, 11 (15.7%) and 16 (45.7%) were managed conservatively; 25 (35.7%) women and nine (25.7%) men were managed with oral medication; 20 (28.6%) women and six (17.1%) men were offered BTX‐A; five (7.1%) women and two (5.7%) men were offered a suprapubic catheter. Of the 16 women with urodynamic SUI, two (7.1%) were offered SUI surgery. SUI without any NDO was observed in 15 women, of which one (6.7%) was offered SUI surgery.

**TABLE 5:** Outcomes based on urodynamic findings.

	*n*	Conservative	α‐Blocker	AC/B3A	Medical	BTX‐A	PTNS	SNM	SPC	SUI surgery	BOO surgery
		*n* (%)	*n* (%)	*n* (%)	*n* (%)	*n* (%)		*n* (%)	*n* (%)		
NDO											
F	70	11 (15.7%)	1 (1.4%)	24 (34.3%)	25 (35.7%)	20 (28.6%)	2 (2.9%)	0 (0.0%)	5 (7.1%)	1 (1.4%)	‐
M	35	16 (45.7%)	2 (5.7%)	7 (20.0%)	9 (25.7%)	6 (17.1%)	0 (0.0%)	0 (0.0%)	2 (5.7%)	0 (0.0%)	1 (2.9%)
T	105	27 (25.7%)	3 (2.9%	31 (29.5%)	34 (32.4%)	26 (24.8%)	2 (1.9%)	0 (0.0%)	7 (6.7%)	1 (1.0%)	‐
LoC											
F	11	6 (54.5%)	1 (9.1%)	2 (18.2%)	3 (27.3%)	2 (18.2%)	0 (0.0%)	0 (0.0%)	0 (0.0%)	0 (0.0%)	‐
M	5	3 (60.0%)	0 (0.0%)	0 (0.0%)	0 (0.0%)	3 (60.0%)	0 (0.0%)	0 (0.0%)	2 (40.0%)	0 (0.0%)	0 (0.0%)
T	16	9 (56.3%)	1 (6.3%)	2 (12.5%)	3 (18.8%)	5 (31.3%)	0 (0.0%)	0 (0.0%)	2 (12.5%)	0 (0.0%)	‐
USUI											
F	28	16 (57.1%)	1 (3.6%)	9 (56.3%)	10 (35.7%)	2 (7.1%)	0 (0.0%)	0 (0.0%)	1 (3.6%)	2 (7.1%)	‐

Abbreviations: AC = anticholinergics; B3A = beta 3 agonist; BOO = bladder outflow obstruction; BTX‐A = botulinum toxin‐A; F = female; M = male; NDO = neurogenic detrusor overactivity; PTNS = posterior tibial nerve stimulation; SNM = sacral neuromodulation; SPC = suprapubic catheter; SUI = stress urinary incontinence; T = total; USUI = urodynamic stress urinary incontinence.

## 4 DISCUSSION

Patients with MS related NLUTD undergoing UDS mainly report storage LUTS and approximately half of the female patients reported SUI. Only 5.1% of women reported pure SUI. Over half of patients underwent UDS before they had tried any medications for OAB. No female patients demonstrated ureteric reflux on VUDS, less than 1% of women had end fill pressures over 40 cmH_2_O although approximately 10% had poor compliance. Approximately 3/4 of patients were offered conservative or oral medication following urodynamics. Very few patients with MS underwent surgery for bladder outflow obstruction or stress incontinence following UDS.

UDS in patients with NLUTD aims to provide an objective diagnosis of the underlying urological pathophysiology to guide treatment of symptoms and to identify any risk factors for upper tract deterioration. Patients with NLUTD are at increased risk of developing renal failure. Those with neural tube defects or spinal cord injury are at 6.8 to 9.0 times more likely to develop renal failure than patients without neurological disease.^21^ Lawrenson et al.'s study did however show that patients with MS have been shown to have a similar risk of developing renal failure as the general population.^21^ While there is no formal definition of an ‘unsafe bladder’, McGuire et al. first reported in 1981 that children with neural tube defects who had detrusor leak point pressures (DLPP) of more than 40 cmH_2_O were at risk of renal deterioration as 68% showed vesico‐ureteric reflux (VUR) and 81% showed ureteric dilatation.^22^ For those with a DLPP less than 40 cmH_2_O, none showed VUR and 10% showed ureteric dilatation. Later studies including those by Ghobash et al. showed that a sustained detrusor pressure over 15 cmH_2_O could lead to upper tract deterioration.^23^ In a consensus document with the centres for disease control, Routh et al. later defined ‘hostile’ bladders as having a DLPP over 40 cmH_2_O, ‘intermediate risk bladders’ 25 to 39 cmH_2_O and safe bladders as those with a DLPP less than 15 cmH_2_O.^24^, ^25^ It is worth bearing in mind that ureteric reflux can dampen bladder pressures giving a false impression of normal compliance in some patients. Although DLPP in the paediatric spina bifida may not be translatable to the adult MS population, the risk of upper tract deterioration was only present in 0.9% female and 6.7% males in our study based on those definitions. This low prevalence of risk factors for upper tract deterioration is the likely reason for the low incidence of renal failure in patients with MS. As such, female patients with MS can be considered ‘low risk’ for upper tract deterioration and UDS do not appear necessary at baseline assessment in keeping with the AUA/SUFU guidelines.^13^ Nevertheless, just over half (51.1%) of female patients reported SUI and only 4.5%reported pure SUI. VUDS does potentially have advantages over standard UDS in these patients as it allows further characterisation of SUI which can help direct management.

Current guidelines for MS are inconsistent.^26^ Despite this, the results of this audit would imply that 57.7% of women underwent UDS before they had tried an anticholinergic or beta 3 adrenoceptor agonist. Following UDS, 27.9% of women were offered conservative treatment, 27% were started on ISC and 4.5% were offered a suprapubic catheter. Many of these patients could have been offered these interventions before UDS, based on the results of non‐invasive investigations. The UK consensus on the management of the bladder in MS and the 2012 NICE guidelines on urinary incontinence in neurological disease would not recommend UDS unless they had tried oral medication^5^, ^12^, ^15^ but would need non‐invasive testing in the form of a renal ultrasound scan to rule out hydronephrosis and check post‐void residuals. Patients have been reported to find UDS mildly painful and embarrassing but tend to accept the test to aid diagnosis.^27^, ^28^ Infection rates following UDS have been reported between 1.6% and 6.2%, though serious complications are rare.^29^, ^30^, ^31^

The findings of our study do not demonstrate whether it would be appropriate to offer intravesical BTX‐A injections to female patients with MS with refractory OAB or urgency predominant UUI, prior to UDS, as it is not an interventional trial. The findings do however show that female patients with MS with rOAB tend not to have high‐risk bladders and it is likely that they will have NDO. It is important for non‐invasive investigations to be done prior to UDS and enable patients to be started on oral medications, be taught intermittent self‐catheterisation, or to be offered a supra‐pubic catheter. Future guidelines should consider recommending standard rather than video urodynamics for female patients with MS, without incontinence, with normal renal function and without a significant post‐void residual volume or hydronephrosis on US scanning.

The study is limited by a relatively heterogenous population regarding self‐catheterisation status, prior treatment and extent of disability. While 84.7% female patients and 87% male patients underwent VUDS, some underwent standard UDS according to individual hospital's policies and availabilities of video fluoroscopy. Further work should focus on how UDS in patients with MS who have failed conservative and medical management affects patient reported treatment outcomes.

## 5 CONCLUSIONS

Over half of female patients underwent UDS before trying conservative and oral medication potentially delaying non‐invasive treatment. Most guidelines only recommend UDS in those being considered for second‐line interventions such as intradetrusor BTX‐A injections or surgery for SUI.

Ureteric reflux and end fill pressures over 40 cmH_2_O were observed in a small but significant number of male patients supporting the need for VUDS rather than SUDS before starting medical or conservative therapy in men with MS. Standard UDS appear to suffice in women with MS who have not responded to conservative measures and oral medication and who report pure OAB symptoms, have normal renal function but do not have hydronephrosis or significant post‐void residuals on ultrasound scanning. Future guidelines should emphasise the importance of non‐invasive investigations for the initial assessment of MS related NLUTD and ensure that VUDS are reserved for those most likely to benefit.

## AUTHOR CONTRIBUTIONS

**Nicholas Faure Walker:** Project Lead, data collection and collation, manuscript preparation and review. **Alexandra Bacon:** Data collection and manuscript review. **Manisha Teji:** Data collection and collation, manuscript preparation and review. **Eskinder Solomon:** Data collection and manuscript review. **Arun Sahai:** Data collection and manuscript review. **Ross Stephens:** Data collection and manuscript review. **Emmanuel Okpii:** Data collection and manuscript review. **Charlotte Dunford:** Data collection and manuscript review. **Sarah Wood:** Data collection and manuscript review. **Emily Speck:** Data collection and manuscript review. **Ruth Doherty:** Data collection and manuscript review. **Angie Rantell:** Data collection and manuscript review. **George Araklitis:** Data collection and manuscript review. **Lydia Lilis:** Data collection and manuscript review. **Tharani Nitkunan:** Data collection and manuscript review. **Michelle Carey:** Data collection and manuscript review. **Suzanne Biers:** Data collection and manuscript review. **Brendan Berry:** Data collection and manuscript review. **Mahreen Pakzad:** Data collection and manuscript review. **Hashim Hashim:** Senior author, data collection and collation,manuscript preparation and review.

## CONFLICT OF INTEREST STATEMENT

Nicholas Faure Walker

- Received charitable donations from Allergan International foundation
- Conference fees and teaching honoraria received from Astellas and Allergan
- PI on Janssen ExPev9V UTI vaccine trial
- PI on Catheter II NIHR trial
- Advisor to MS Society
- ICS standardisation steering committee member

George Araklitis

- Received speaker fees from Medtronic

Suzanne Biers

- AbbVie have provided an educational visit/grant to attend the EAU 2025

Hashim Hashim

- Speaker for Medtronic and Astellas
- Committee member of EAU male LUTS guidelines
- Committee member of BAUS‐FRU Executive committee
- Associate Editor of BJUI Compass and Neurourology and urodynamics

Angie Rantell

- Advisory boards/Panels/honoraria for teaching for Convatec, Holister, Coloplast, Laborie, Essity, B Braun, Mediplus

Arun Sahai

- Speaker fees, trials, unrestricted educational

The remaining authors have no conflicts of interest.
